# Naringenin Modifies the Development of Lineage-Specific Effector CD4^+^ T Cells

**DOI:** 10.3389/fimmu.2018.02267

**Published:** 2018-10-01

**Authors:** Junpeng Wang, Xinli Niu, Chunfang Wu, Dayong Wu

**Affiliations:** ^1^Institute of Infection and Immunity of Huaihe Hospital, Henan University, Kaifeng, China; ^2^College of Life Science, Henan University, Kaifeng, China; ^3^Jean Mayer USDA Human Nutrition Research Center on Aging, Tufts University, Boston, MA, United States

**Keywords:** Naringenin, CD4^+^ T cells, T cell subsets, cell differentiation, autoimmune diseases

## Abstract

Disrupted balance in the lineages of CD4^+^ T cell subsets, including pro-inflammatory T helper (Th) cells and anti-inflammatory regulatory T cells (Treg), is a primary pathogenic factor for developing autoimmunity. We have found that this immunomodulatory effect of naringenin on effector T cells and T-cell mediated experimental autoimmune encephalomyelitis (EAE). We therefore explored the effects of naringenin on the development of different effector CD4^+^ T cells. Naïve CD4^+^ T cells were differentiated under respective Th1, Th2, Th17, and Treg polarizing conditions with naringenin. Percent populations of each differentiated CD4^+^ T cell subsets were determined and the corresponding regulating pathways were investigated as underlying mechanisms. Naringenin mainly inhibited CD4^+^ T cell proliferation and differentiation to Th1 and Th17, but did not affect Th2 cells. Impeded Th1 polarization was associated with inhibition of its specific regulator proteins T-bet, p-STAT1, and p-STAT4 by naringenin. Likewise, Th17 regulator proteins RORγt, p-STAT3, and Ac-STAT3 were also inhibited by naringenin. In addition, naringenin promoted Treg polarization and also prevented IL-6-induced suppression of Treg development via down-regulation of p-Smad2/3 as well as inhibition of IL-6 signaling, and the latter was further supported by the *in vivo* results showing lower soluble IL-6R but higher soluble gp130 levels in plasma of naringenin-fed compared to the control EAE mice. Naringenin impacts CD4^+^ T cell differentiation in a manner that would explain its beneficial effect in preventing/mitigating T cell-mediated autoimmunity.

## Introduction

Naïve CD4^+^ T cells can differentiate into distinct effector helper T cell (Th) subsets, including Th1, Th2, and Th17 cells, as well as regulatory T cells (Treg) (1–3). Th1, Th17, and Treg cell subsets have been regarded as major players in immunopathology of autoimmune diseases. Th1 and Th17 cells are pro-inflammatory subsets that promote the development of autoimmunity and tissue damage, while Treg cells maintain immunotolerance and prevent autoimmunity. Thus, maintaining the balance of anti-inflammatory Treg cells and pro-inflammatory Th1 and Th17 cells has significant implication in preventing and/or attenuating autoimmunity and chronic inflammation.

Protective, nonpathogenic Th1 and Th2 cells can be generated *in vitro* from naïve T cells by using IL-12 and IL-4 which is regulated by their specific transcription factors T-bet and GATA3, respectively (4, 5).The cytokine TGF-β drives the conversion of naïve T cells into induced Treg (iTreg) cells, while TGF-β, together with pro-inflammatory cytokines, in particular IL-6, drives naïve CD4^+^ T cell differentiation toward Th17 (3, 6). Mechanistically, TGF-β alone can activate its downstream transcription factors Smad2 and Smad3 to induce expression of Treg-specific marker Foxp3, which control the generation and function of Treg. In contrast, IL-6 induces activation of STAT3 to promote expression of Th17 cell-specific transcription factor RORγt critical for IL-17 expression.

Furthermore, TGF-β-induced Foxp3 suppressed RORγt function partly via their interaction (7). Therefore, the fate of naïve CD4^+^ T cells upon stimulation by antigens to turn into Th17 or Treg cells for a significant part depends on the micro-environmental cytokine-regulated balance of RORγt and Foxp3.

Naringenin, a major flavanone in grapefruits, has a wide range of anti-inflammatory and neuro-protective properties (8). We recently reported that dietary naringenin supplementation ameliorated experimental autoimmune encephalomyelitis (EAE) in mice, which was associated with the decrease in Th1 and Th17 cell populations and pro-inflammatory cytokine IL-6 production, which promotes CD4^+^ T cells differentiation into Th17 cells (9). In addition, our *in vitro* study showed that naringenin directly inhibited effector T cell functions, including T cell proliferation, cell division, and production of cytokines IL-6, IFN-γ, and IL-17, in normal and EAE mice (10). These data suggest that naringenin may affect CD4^+^ T cell differentiation process. However, there was no direct evidence to substantiate this hypothesis and furthermore, if it is the case, it would be important to know through what molecular mechanisms naringenin exerts its such effect. Thus, in the present study, using *in vitro* model, we characterized (1) which type of T cells (CD4^+^ or CD8^+^) are affected by naringenin, and (2) how naringenin modulates CD4^+^ T cell differentiation into effector lineages (Th1, Th17, and Treg), and (3) what regulating networks are involved in the effects of naringenin on regulating CD4^+^ T cell differentiation.

## Materials and methods

### Animals

Specific pathogen-free C57BL/6 female mice (6–8 wk) were purchased from Nanjing Biomedical Research Institution of Nanjing University (Nanjing, China). Mice were maintained at a controlled environment with a 12 h light:dark cycle and provided *ad libitum* access to water and mouse chow. Mice were killed by CO_2_ asphyxiation followed by exsanguination and tissues were collected post-mortem. All conditions and handling of the animals were approved by the Institutional Animal Care and Use Committee of Huaihe Hospital at Henan University.

### T cell division

After mice were euthanized, inguinal lymph node (LN) cells were collected and single cells suspension was prepared for evaluation of CD4^+^ and CD8^+^ T cell proliferation using tracking dye fluorescein diacetatesuccinimidyl ester (CFSE, Molecular Probes, Eugene, OR, USA) method as previously described (10). A stock solution of naringenin (Sigma-Aldrich, St. Louis, CA) dissolved in DMSO at 400 mM was stored at −80°C and diluted with culture medium to the appropriate working concentrations immediately prior to use. Briefly, after LN cells were labeled with 1 μM of CFSE, they were added to a 24-well plate at 2 × 10^6^/well and stimulated with immobilized anti-CD3 Ab at 5 μg/ml and soluble anti-CD28 Ab at 1 μg/ml (anti-CD3/CD28) (both from Biolegend, San Jose, CA) in the presence of different levels of naringenin for 48 h. At the end of incubation, cells were collected, washed, and stained with fluorochrome conjugated anti-CD3, anti-CD4, and anti-CD8 (eBioscience). Fluorescence signals of stained cells were acquired by an Accuri C6 (Ann Arbor, MI) flow cytometer and data were analyzed with FlowJo7.6 software (Treestar Inc., OR, USA).

### Intracellular cytokine measurement

After spleen cells were stimualted with anti-CD3/CD28 in the presence of naringenin for 48 h, they were re-stimulated during the last 4 h with 50 ng/ml PMA and 500 ng/ml ionomycin (both from Sigma-Aldrich) in the presence of monensin (GolgiStop, BD Pharmingen, San Jose, CA), and then the frequency and intensity of IFN-γ and IL-4 in CD4^+^ and CD8^+^ T cells were performed using flow cytometry method as described above.

### CD4^+^ T cell differentiation

Naïve CD4^+^ T cells were isolated from spleens using a CD4^+^CD62L^+^ T cell isolation kit II (Miltenyi Biotec, Auburn, CA) and incubated at 2 × 10^6^ cells/ml completed RPMI-1640 medium containing 5%FBS in 24-well plate. Cells were activated with anti-CD3/CD28 in all the experiments described below and T cell differentiation was induced as described previously (11). Briefly, the cultures were supplemented with IL-12 (10 ng/ml) (R&D systems, Inc., Minneapolis, MN) and anti-IL-4 (10 μg/ml) (BD Pharmingen) for Th1 differentiation, with IL-4 (10 ng/ml) (R&D systems, Inc.) and anti-IFN-γ (10 μg/ml) (BD Pharmingen) for Th2 differentiation, and with IL-6 (20 ng/ml), TGF-β (5 ng/ml), IL-23 (20 ng/ml) (all from R&D system), anti-IFN-γ, and anti-IL-4 (each 10 μg/ml) for Th17 differentiation. For Treg differentiation, naïve CD4^+^ T cells were incubated in the presence of TGF-β (5 ng/ml) for 72 h. To determine if naringenin (80 μM) affects the reciprocal effect between Treg and Th17, IL-6 was also added during Treg differentiation. Intracellular levels of Th1 (IFN-γ), Th2 (IL-4, IL-10, and IL-13), Th17 (IL-17A), and Treg (Foxp3) (all from eBioscience) were determined by flow cytometry as previously described (11). In addition, differentiated cells were stained with fluorochrome-conjugated anti-STAT1 (pY701/p-STAT1), anti-STAT3 (pY705/p-STAT3), anti-STAT4 (pY693/p-STAT4), T-bet (all from BD Pharmingen), and RORγt (R&D systems, Inc.) following standard protocols as described previously (11). Isotype Controls were used as negative control. Cells were analyzed using flow cytometry as described above.

### Western blot

Naive CD4^+^ T cells were cultured with anti-CD3/CD28 and TGF-β with/without IL-6 in the presence/absence of naringenin for the time as indicated in the result section. Cells were harvested at 3 × 10^6^ cells/50 μl into RIPA cell lysis buffer containing 50 mM Tris-HCl (pH 7.4), 150 mM NaCl, 1% NP40, 1 × protease inhibitor cocktail (Roche Applied Science, Indianapolis, IN), and 1 × phosphatase inhibitor cocktail (Sigma-Aldrich), and incubated on ice for 15 min. Total cell protein extract was resolved in 7.5% acrylamide gels and then transferred to nitrocellulose membranes. The membrane was blocked with 5% non-fat milk in Tris-buffered saline before being incubated, respectively with specific primary antibodies for the following proteins: STAT-3(1:1000), Smad2/3 (1:1000), phosphorylated Smad2/3 (p-Smad2/3) (1:1000), Acetyl-STAT3 (Lys685, Ac-STAT3) (1:1000), phosphorylated STAT3 (p-STAT3) (1:1000) (all from Cell Signaling Technologies, Danvers, MA), and β-actin (1:5000, Sigma-Aldrich). The membranes were next incubated with horseradish peroxides (HRP)-conjugated secondary antibodies followed by exposure to enhanced chemiluminescent reagents (Millipore, Burlington, MA).

### Induction and evaluation of EAE

Mice were fed a diet supplemented with 0.5% naringenin and then immunized to induce EAE as described before (9). At day 42, mice were killed, and plasma was collected to measure soluble IL-6Rα (sIL-6R) and soluble gp130 (sgp130) as described below.

### Detection of mIL-6R, mgp130, sIL-6Rα and sgp130

After differentiation, one set of cells was stained with fluorochrome-conjugated anti-CD4, anti-CD126 (mIL-6R), and anti-CD130 (mgp130) (all from eBioscience). Cells were analyzed using flow cytometry as described above in “T cell division.”

The concentrations of sIL-6Rα and sgp130 in the plasma samples mentioned above or supernatants from differentiated CD4^+^ T cells were quantified using the IL-6R and gp130 ELISA kit (Sino Biological Inc., Beijing) following the manufacturer's instruction.

### IL-6-induced phosphorylation and acetylation of STAT3

After naïve CD4^+^ T cells were incubated with/without 80 μM naringenin for 2 h, recombinant IL-6 (20 ng/ml) was added to the cultures and incubated at 37°C in a water bath for 15 min. Total cell protein was extracted and the p-STAT3 and Ac-STAT3 levels were determined as described above in “Western blot.”

### Statistical analysis

Results are expressed as means ± SD. Statistical analysis was conducted using SYSTAT 12 statistical software. Differences were determined using one-way ANOVA followed by Tukey's HSD *post-hoc* test for multiple comparisons, or non-paired Student's *t*-test. Significance was set at *P* < 0.05.

## Results

### Naringenin impacts CD4^+^ T cell functions

**Figure 1:**
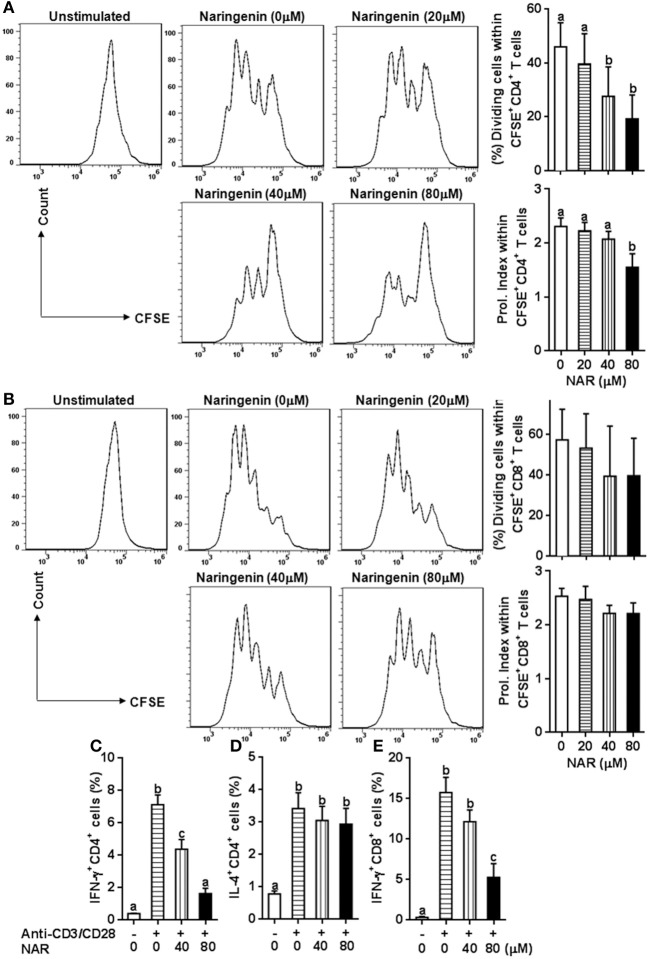
Effect of Naringenin on CD4^+^ and CD8^+^ T cell proliferation and cytokine production. Cell division and proliferation index of CD3^+^CD4^+^ **(A)** and CD3^+^CD8^+^ T **(B)** cells was determined from anti-CD3/CD28-activated CFSE-labeled LN cells with different level of naringenin by flow cytometry. In addition, the proportion of CD4^+^ T cell-secreting IFN-γ **(C)** and IL-4 **(D)**, and CD8^+^ T cell-secreting IFN-γ **(E)** was determined from anti-CD3/CD28 LN cells using flow cytometry. *Histogram figures* are representative results, and bar figures are mean ± SD of three independent experiments. Means without a common letter significantly differ at least at *P* < 0.05. NAR, naringenin; Prol. Index, proliferation index.

We in a previous study found that naringenin inhibited T cell proliferation in anti-CD3/CD28-activated lymphocytes (10). However, it is still unclear how different types of T cell populations (CD4^+^ and CD8^+^) may be affected by naringenin. Thus, we first evaluated CD4^+^ and CD8^+^ T cell proliferation from anti-CD3/CD28-activated lymphocytes treated by naringenin. As shown in Figures 1A,B, naringenin dose-dependently inhibited CD4^+^ T cell division and proliferation index, but did not significantly affect CD8^+^ T cells. Furthermore, naringenin inhibited CD4^+^ T cell production of IFN-γ (Th1 response) (Figure 1C), but not IL-4 (Th2 response) in a dose-dependent manner (Figure 1D). While the high concentration of naringenin (80 μM) decreased IFN-γ production in CD8^+^ T cells stimulated by anti-CD3/CD28 (Figure 1E). These data suggest that naringenin mainly affects CD4^+^ rather than CD8^+^ T cells; within CD4^+^ T cells, it appears that Th1 rather than Th2 response is affected by naringenin.

### Naringenin inhibits Th1, but not Th2 differentiation

**Figure 2:**
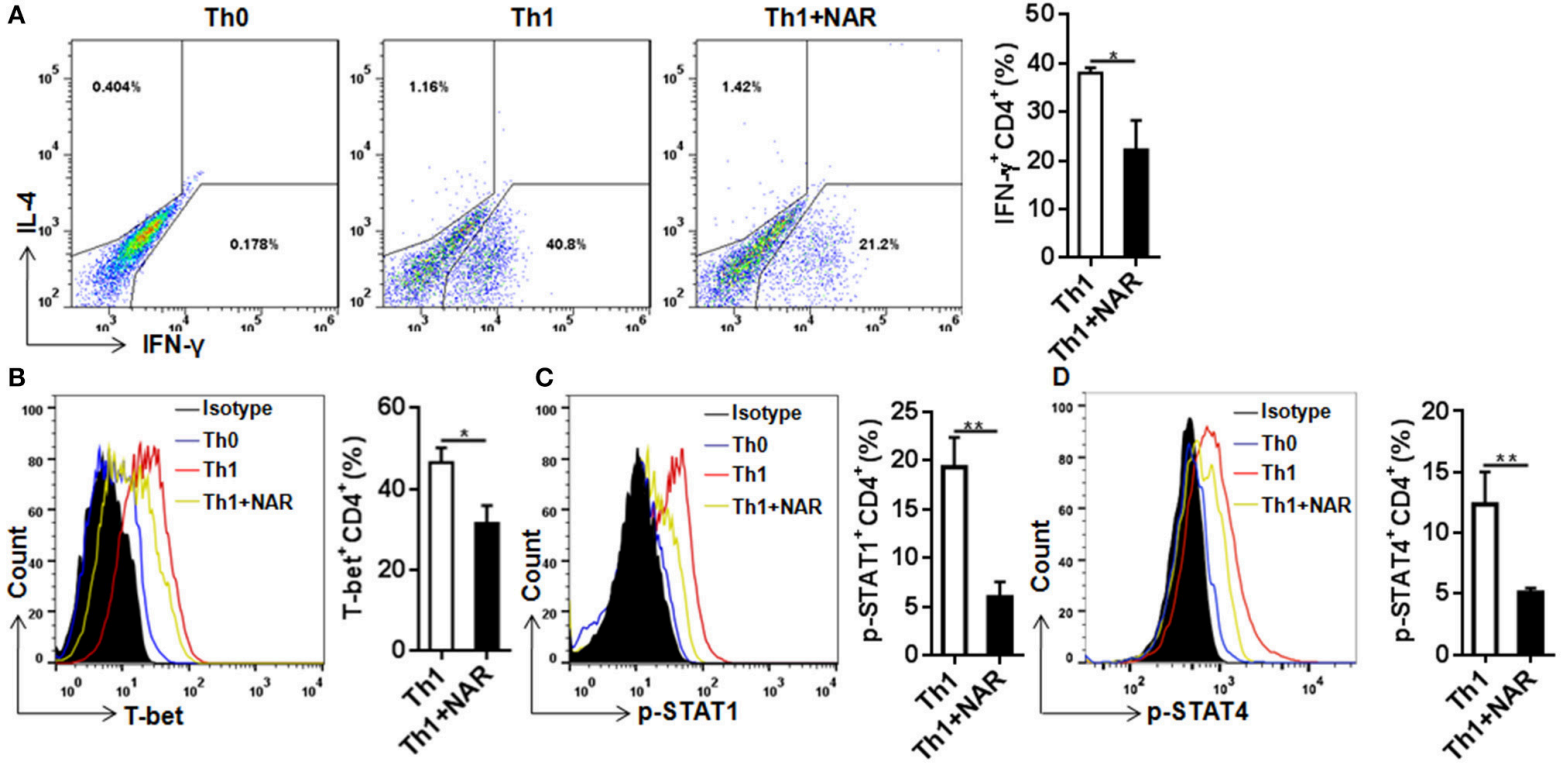
Naringenin inhibits Th1 differentiation via affecting the corresponding regulation network. Naïve CD4^+^ T cells from C57BL/6 mice were activated with anti-CD3/CD28 under Th1-polarizing condition with or without 80 μM naringenin. Intracellular level of IFN-γ **(A)**, T-bet **(B)**, p-STAT1 **(C)**, and p-STAT4 **(D)** in differentiated CD4^+^ T cells was evaluated by flow cytometry. *Dot scatters* and *histogram* figures is representative results, and bar figures are mean ± SD of three independent experiments. ^*^P < 0.05 and ^**^P < 0.01 by Student's *t-*test. NAR, naringenin.

**Figure 3:**
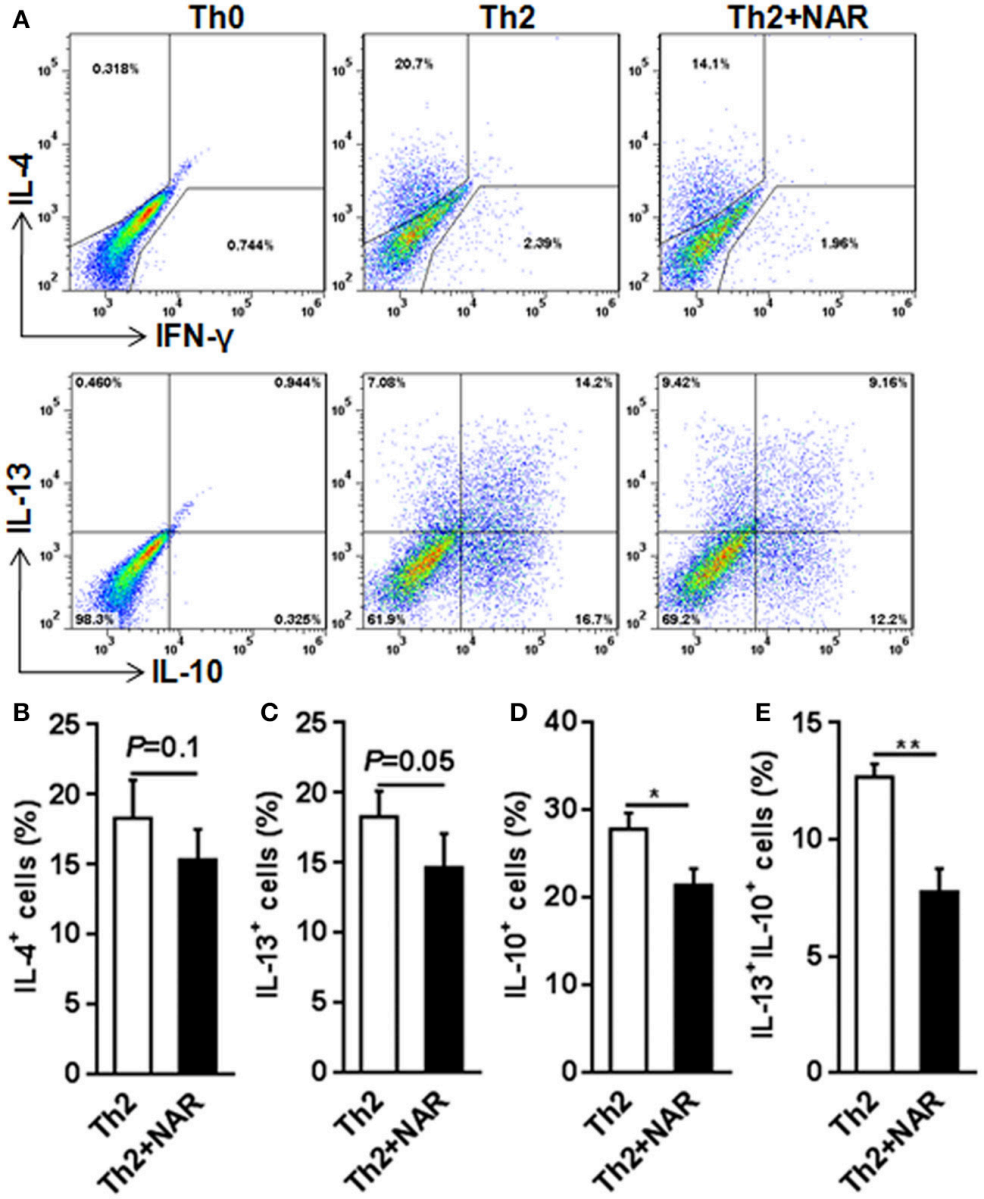
Effect of naringenin inhibits Th2 differentiation. Naïve CD4^+^ T cells from C57BL/6 mice were activated with anti-CD3/CD28 under Th2-polarizing condition with or without 80 μM naringenin. Intracellular level of IL-4 **(A, B)**, IL-13 **(C)**, IL-10 **(D)**, and IL-13^+^IL-10^+^ **(E)** in differentiated CD4^+^ T cells was evaluated by flow cytometry. *Dot scatters* is representative results, and bar figures are mean ± SD of three independent experiments. ^*^P < 0.05 and ^**^P < 0.01 by Student's *t*-test. NAR, naringenin.

Our previous *in vivo* study showed that EAE mice receiving naringenin had smaller Th1 cell population, but similar Th2 cell population compared to those fed control diet (9). However, since the magnitude of a given cell population in the body may be affected by multiple factors including proliferation, differentiation, and shrinking, as well as interaction among different cell population, we speculated but could not convincingly conclude that naringenin directly affects CD4^+^ T cell differentiation. Thus to seek direct answer to this issue, in the current study we used an *in vitro* differentiation model, in which naive CD4^+^ T cells cultured under standard Th1 or Th2 polarization condition and production of IFN-γ and IL-4 was used as hallmark for Th1 and Th2, respectively. We found that Th1 polarization was inhibited by naringenin (80 μM) compared to the control (21 vs. 40%) (Figure 2A); while Th2 polarization was not significantly affected by naringenin (Figures 3A,B). These results are in agreement with those in the *in vivo* study. Additionally, we found that in Th2-polarized CD4^+^ T cells, IL-13^+^ population was marginally decreased (*P* = 0.05) (Figure 3C) and IL-10^+^ (Figure 3D) and IL-10^+^IL-13^+^ (Figure 3E) populations were significantly decreased by naringenin.

To further investigate how naringenin modulated regulation mechanism upstream to Th1 differentiation, we determined expression of T-bet, a transcriptional factor known to be the master regulator in Th1 cell differentiation. Consistent with naringenin's inhibitory effect on Th1 differentiation, naringenin was found to decrease T-bet expression in differentiating CD4^+^ T cells (Figure 2B). It has been shown that T cell differentiation toward Th1 subset can be triggered by IL-12 and IFN-γ signaling via their transducers STAT4 and STAT1, respectively, which induce T-bet expression and drive Th1 cell differentiation (12, 13). Thus, we next determined involvement of STAT activation. Indeed, STAT1 and STAT4 activation (phosphorylation) in CD4^+^ T cells cultured under Th1 polarization condition was inhibited by naringenin treatment (Figures 2C,D).

### Naringenin inhibits Th17 cell differentiation

**Figure 4:**
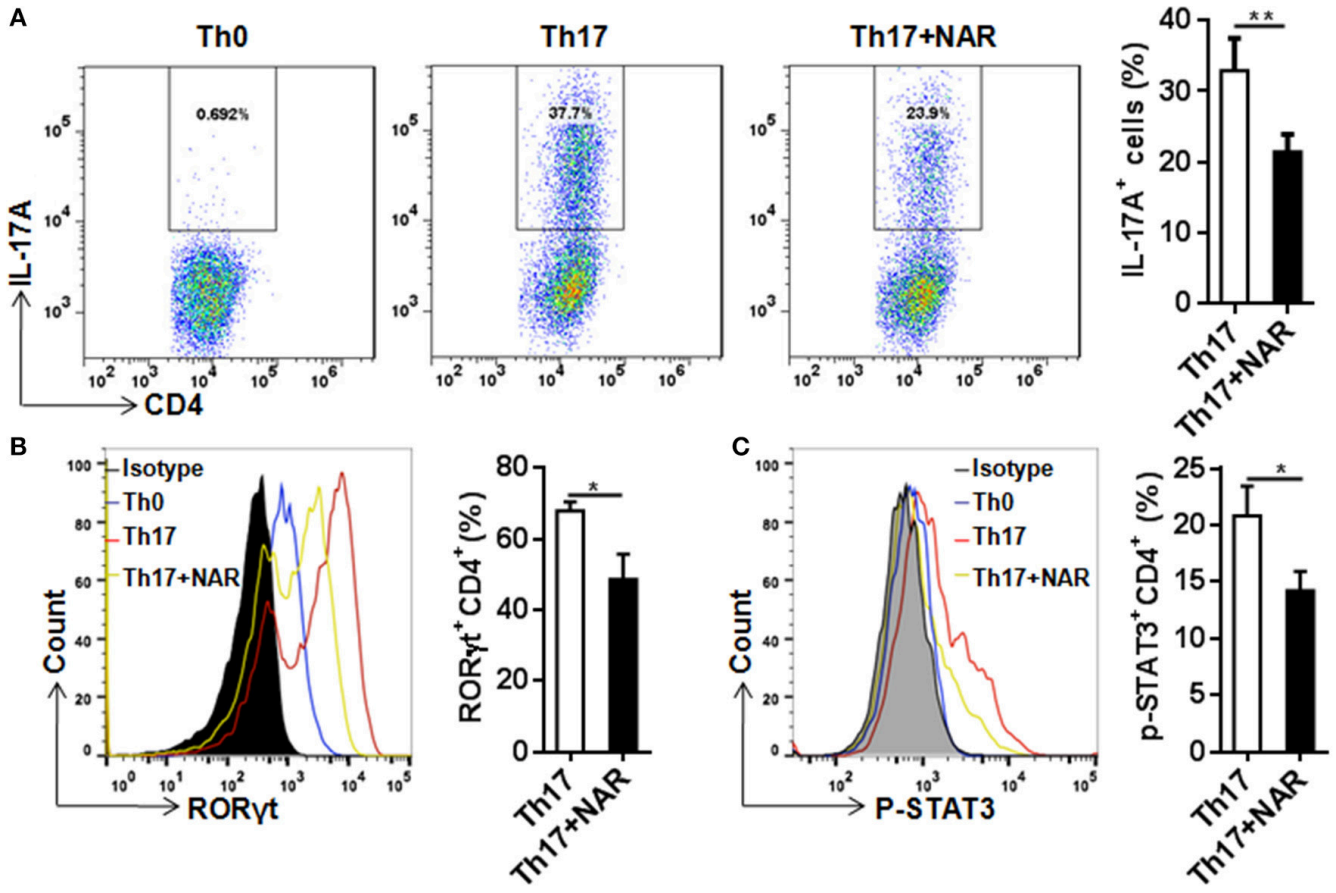
Naringenin inhibits Th17 differentiation via affecting the corresponding regulation network. Naïve CD4^+^ T cells from C57BL/6 mice were activated with anti-CD3/CD28 under Th17-polarizing condition with or without 80 μM naringenin. Intracellular level of IL-17 (A), RORγt (B), and p-STAT3 (C) in differentiated CD4^+^ T cells was evaluated by flow cytometry. *Dot scatters* and *histogram* figures is representative results, and bar figures are mean ± SD of three independent experiments. ^*^P < 0.05 and ^**^P < 0.01 by Student's *t-*test. NAR, naringenin.

Th17 cells, which are commonly defined as IL-17-producing CD4^+^ T cells, are present at low level in naïve T cells. In the autoimmune disorders such as EAE, this population can be greatly increased. Th17 cells are believed to play a critical role in the development of autoimmunity. Under the *in vitro* polarizing conditions, naïve CD4^+^ T cells can be driven to develop into Th17 cells, usually by TCR stimulation in the presence of IL-6 and TGF-β. In such an experimental setting, we found that naringenin prohibited differentiation of naïve CD4^+^ T cells into IL-17-producing Th17 cells (Figure 4A).

RORγt is a specific transcription factor driving Th17 cell differentiation, and STAT3 is the key signal transducer which mediates action of IL-6, IL-21, and IL-23 (14, 3). Consistent with its effect on Th17 differentiation, nairngenin inhibited RORγt expression (Figure 4B) as well as its upstream event, STAT3 phosphorylation (Figure 4C).

### Naringenin promotes iTreg development and prevents IL-6-induced suppression of treg development

**Figure 5:**
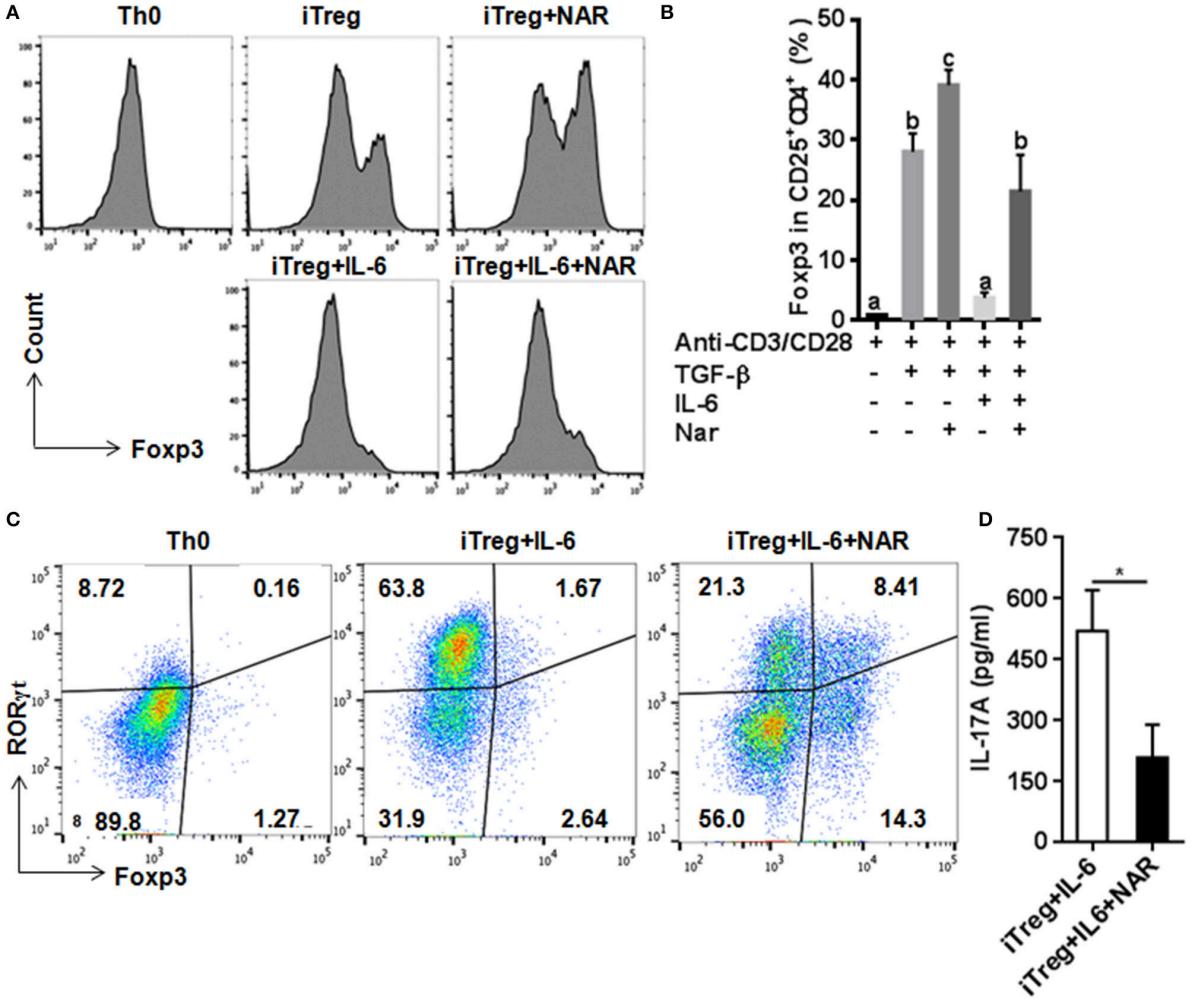
Naringenin promotes iTreg differentiation and prevented IL-6-induced suppression of iTreg differentiation. Naïve CD4^+^ T cells from C57BL/6 mice were activated with anti-CD3/CD28 under iTreg-polarizing condition with or without IL-6 with or without 80 μM naringenin. Effect of naringenin on the Foxp3 expression under iTreg-polarizing condition was determined by flow cytometry **(A)**. Intracellular levels of RORγt and Foxp3 expression **(B)** and the IL-17A level (C) from cell-free supernatants were determined under iTreg-polarizing condition with IL-6. *Histogram* figures and *dot scatters* are representative results, and bar figures are mean ± SD of three independent experiments. Means without a common letter significantly differ at least at *P* < 0.05. ^*^P < 0.01 by Student's *t*-test. NAR, naringenin.

**Figure 6:**
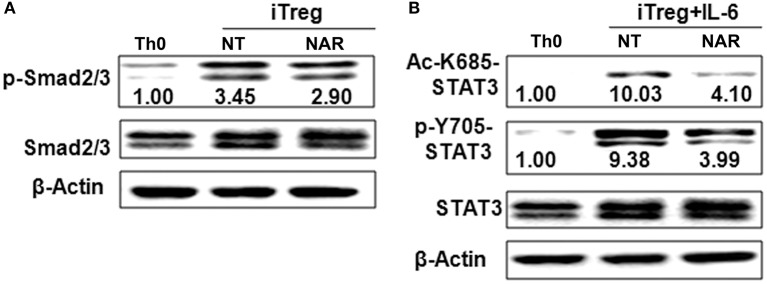
Naringenin affects Smad2/3 and STAT3 activation under iTreg-polarizing condition with or without IL-6. Naïve CD4^+^ T cells from C57BL/6 mice were activated with anti-CD3/CD28 under iTreg-polarizing condition with or without IL-6 with or without 80 μM naringenin. Effect of naringenin on phosphorylated Smad2/3 expression under iTreg-polarizing condition was determined by Western blot **(A)**. Phosphorylation and acetylation of STAT3 expression were determined by Western blot under iTreg-polarizing condition with IL-6 **(B)**. The gel pictures are representatives of three independent experiments, which had similar results. The values below band images are the ratios of p-Smad2/3, p-STAT3/STAT3, and Ac-STAT3/STAT3. NAR, naringenin.

Our *in vivo* study showed that dietary naringenin did not affect Treg cells in EAE mice (9). Varied results have been reported regarding how naringenin affects Treg development (15, 16). It is noted that those results were generated with use of different culture conditions, which often makes data interpretation difficult. Thus, we next determined whether naringenin affects iTreg development under standard iTreg-polarized condition. Although naringenin promoted Treg cell differentiation driven by TGF-β (Figure 5A), it only moderately reduced activation of Smad2/3 (3.45 vs. 2.90), a transducer of TGF-β-mediated Foxp3 induction (Figure 6A).

IL-6 has been shown to inhibit Treg cell generation induced by TGF-β which has been demonstrated in this study and our previous study (11). This IL-6-induced inhibition of Treg development was prevented by naringenin (Figure 5A). This is consistent with reported decrease in Th17 differentiation by naringenin because the presence of IL-6 can switch TGF-β-induced differentiation in favor of Th17, and there is reciprocal modulation between Treg and Th17 differentiation. To further confirm this, we also directly determined Th17 population in the same cultures in which Treg were quantified and found that naringenin caused a significant reduction of Th17 population as manifested by decreased the level of both RORγt and IL-17 (Figures 5B,C). As a further support, it was also found that naringenin decreased p-STAT3 and Ac-STAT3 expression under TGF-β plus IL-6 co-cultured condition (Figure 6B).

### Naringenin interferes with IL-6/IL-6R signaling to affect treg cell development

**Figure 7:**
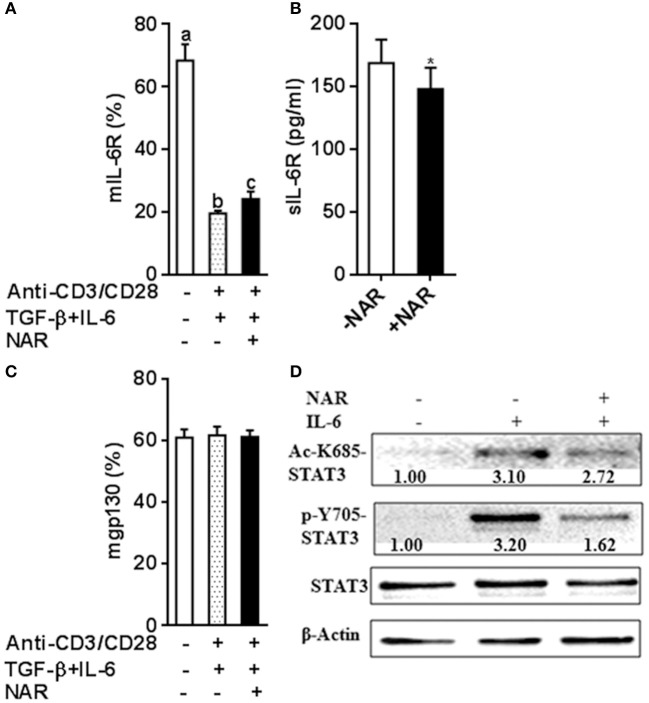
Naringenin affects IL-6 receptor expression under iTreg-polarizing condition with IL-6 and inhibits IL-6 downstream signaling. **(A–C)**, Naïve CD4^+^ T cells from C57BL/6 mice were activated with anti-CD3/CD28 under iTreg-polarizing condition with IL-6 in the absence or presence of 80 μM naringenin. mIL-6R and mgp130 expression was determined by flow cytometry and sIL-6R levels in the cultured medium were quantified by ELISA. Values are mean ± SD of three independent experiments. Means without a common letter significantly differ at least at *P* < 0.05. ^*^P < 0.05 compared with the corresponding control (without NAR) by student's *t*-test. D, After naïve CD4^+^ T cells were incubated with 80 μM naringenin for 2 h, IL-6 was added to stimulate cells for 15 min. Phosphorylation and acetylation of STAT3 expression were determined by Western blot. The gel pictures are representatives of three independent experiments, which had similar results. The values below band images are the ratios of Ac-STAT3/STAT3. NAR, naringenin.

It has been known that IL-6 conveys signals through STAT3 to promote Th17 and inhibit Treg lineage commitment. Classical IL-6R signaling and IL-6 *trans*-signaling have been involved in inflammatory diseases (17, 18). Naïve T cells have high mIL-6R expression and then are lost during an immune response that becomes an important source of sIL-6R (19, 20). IL-6 or IL-6R cannot bind to gp130 alone. A complex of IL-6-IL-6R is necessary for binding to gp130 to form a high-affinity, signaling-competent hexamer that activates STAT3 induces RORγt expression but not Foxp3 expression induced by TGF-β (21, 22). We thus hypothesized that naringenin may influence Th17/Treg balance through modulating IL-6 signaling. To address this, we first evaluated IL-6R expression under Th17/Treg polarized conditions in the absence of neutralized conditions. In accordance with our and other previous reports (11, 20), naïve unstimulated CD4^+^ T cells had high mIL-6R expression and sIL-6R was undetected in cultured medium alone in the absence of anti-CD3/CD28 Abs. The mIL-6R expression (Figure 7A) decreased and the levels of sIL-6R increased upon activation (Figure 7B), both of which were partially prevented by naringenin. We further showed that expression of another IL-6R, mgp130, was not affected by naringenin treatment (Figure 7C). Finally, we found that naringenin diminished IL-6-induced STAT3 phosphorylation and acetylation, the indicator downstream events in IL-6 signaling (Figure 7D). Together, these data suggest that naringenin-induced alteration in IL-6 signaling may be an important mechanism for its effect on Th17/Treg balance.

### Results from dietary supplementation study support naringenin's inhibitory effect on IL-6 signaling

**Figure 8:**
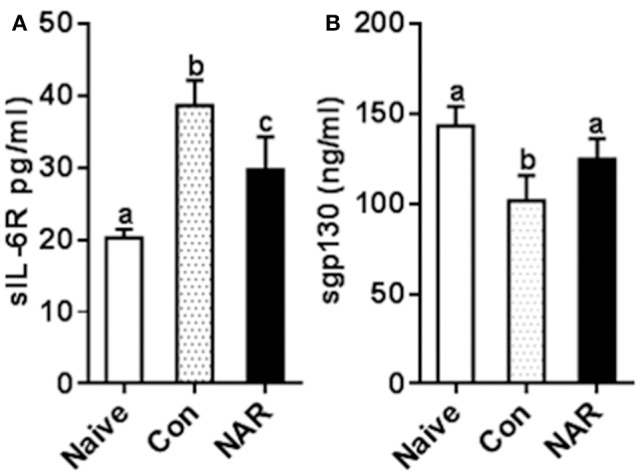
Figure 8 Naringenin reduces level of sIL-6R while increasing sgp130 level in the plasma of EAE mice. C57BL/6 mice were fed a diet containing 0.5% naringenin for 30 day before they were immunized to induce EAE as previously described (10). On day 14 post-immunization, mice were euthanized, and plasma was collected to determinesIL-6R **(A)** and sgp130 **(B)** in the plasma levels. Values are mean ± SD (12/group). Means without a common letter significantly differ at least at *P* < 0.05. Con, control; NAR, naringenin.

To test whether the *in vitro* study results were relevant to the *in vivo* situation, we measured plasma sIL-6R and sgp130 concentrations utilizing the samples collected from naringenin-fed mice in our previous study which showed the attenuated EAE mice by naringenin. We found that sIL-6R was higher and sgp130 was lower in plasma from EAE mice compared to that from the naïve (unimmunized normal) mice, and these changes were partially prevented by dietary naringenin supplementation (Figure 8). These *in vivo* results validate our *in vitro* results in terms of naringenin's effect on IL-6 signaling.

## Discussion

A delicate balance between effector T cells with different functions, in particular pro-inflammatory and pro-tolerance, plays a crucial role for eliciting protective immune response to pathogens without losing immune tolerance to self-antigens. Failure to maintain this balance is an important mechanism responsible for the development of many autoimmune diseases. Therefore, exploring the new strategies targeting this factor should have significant clinical potential in dealing with autoimmune diseases. Targeted drug therapy has made impressing progress; however, the efficacy vs. side effect is still a major issue limiting unrestricted application. Nutritional intervention through consuming bioactive food components has become a desirable alternative and complementary strategy for this purpose. Several major categories of dietary flavonoids are known to have immune-modulating property, which implies their potential application in preventing and/or mitigating autoimmune diseases. We recently showed that dietary supplementation with naringenin, a flavonoid compound found abundant in citrus fruits, particularly in grapefruit, attenuated EAE symptoms and pathology via favorably modulating effector T cell functions involved in T cell-mediated autoimmunity (9). In an *in vitro* study we further demonstrated that naringenin could directly suppress effector T cell functions including total T cell proliferation, and production of cytokines (10). In this study, we demonstrated that naringenin primarily affected functions of effector CD4^+^ cells, which is based on the following observations: (1) T cell proliferation and IFN-γ production in CD4^+^ T cells were inhibited by naringenin; (2) under Th1 differentiation condition, naringenin not only diminished Th1differentiation, but also decreased Th1-specific transcription factor T-bet and transducer STAT4 (for IL-12) and STAT1 (for IFN-γ) activation; (3) naringenin impaired Th17 differentiation which might be mediated by the down-regulation of RORγt, p-STAT3, and Ac-STAT3 under Th17 differentiation condition; (4) naringenin promoted iTreg cells under iTreg polarization condition via down-regulating Smad2/3 phosphorylation; (5) under iTreg polarization condition in the presence of IL-6, naringenin prevented IL-6-induced iTreg suppression through suppressing IL-6/IL-6R signaling, and STAT3 phosphorylation and acetylation. Together, these results suggest that naringenin plays a crucial role in maintaining the balance between Treg and pro-inflammatory T helper cells, which sheds light on the mechanistic insight to its beneficial effect observed in our previous study.

The immune system has evolved several mechanisms to control activated T cell expansion and differentiation, including anergy, death, and regulation (23). One level of control resides in the function of CD4^+^and CD8 ^+^ regulatory cells (24, 25). While CD4^+^ T cells are primary cells in mediating adaptive immunity to a variety of pathogens, they are also a key player implicated in regulation of autoimmunity by their pro-inflammatory and pro-tolerance functions (2). Likewise, CD8^+^ T cells are important in effective vaccination and vial clearance as well as participant in maintaining the immune-tolerance (26–28), On the flip side, however, CD8^+^ T cells are the effector cells contributing to the disease of autoimmunity (29–31). Therefore, altered control of CD4^+^ and CD8^+^ T cells in their response to self-Ag is expected to significantly impact outcomes of autoimmune diseases. We recently reported that naringenin was an inhibitor of effector function of T cells (10). In this study, we expanded the research along this line by investigating how naringenin affected T cell sub-populations because of their unique function and implication in the development of autoimmune disease. We showed, for the first time, that naringenin mainly affected CD4^+^ T cell proliferation, among which naringenin inhibited Th1 response, but had no effect on Th2 response. Although CD8^+^ T cell proliferation was not significantly inhibited by naringenin with the concentrations used, IFN-γ production from CD8^+^ T cells appeared to be dose-dependently inhibited and this effect was clearly significant at high level of naringenin (80 μM). Given this, whether naringenin-induced change in CD8^+^ T cell functions has significant contribution to its beneficial effect in autoimmunity remains to be further investigated.

Although CD8 ^+^ T cells have been shown to be involved in the development of autoimmune diseases such as MS and EAE (31, 26), it is generally accepted that over-activation of self-Ag pathogenic CD4^+^ T cells is the direct cause of these diseases (3, 32). IFN-γ-secreting Th1 cells (33) and IL-17-secreting Th17 cells (3) are first primed in the periphery, migrate into central nervous system (CNS), and then cause demyelization and neurological disability (34). Th1 and Th17 cells can also help recruit other inflammatory cells into CNS to exacerbate the disease process (3). Thus, the agents which target to pro-inflammatory Th1 and Th17 cell populations should be taken as potential candidates for preventing and/or treating autoimmune diseases like MS. Our recent *in vivo* study demonstrated that dietary naringenin reduced immune cell infiltration, and attenuated demyelination in CNS, and these changes were associated with decreased Th1 and Th17 cells, which were, in turn, associated with down-regulation of their respective transcription factors, T-bet and RORγt. However, anti-inflammatory Treg cells were not found to be affected by naringenin (9). These results suggest that naringenin may modulate CD4^+^ T cell subset balance via directly impacting their differentiation processes. Indeed, in the current study, we provided direct evidence supporting this hypothesis as we found that naringenin decreased differentiation of naive CD4^+^ T cells into Th1 and Th17 cells, while increased Treg cell differentiation. In addition, naringenin-induced alteration in CD4^+^ T cell subsets might be due in part to its specific effect on the reduction in abundance or activity of the corresponding regulators for each sunset. These findings reinforce our understanding of beneficial effect of naringenin for the management of autoimmune diseases, which contribute to developing the effective preventive and/or therapeutic approach to combat T-cell mediated autoimmune response.

Treg cells play an important role in maintaining immune tolerance against self-tissues. Some compounds such as TGF-β (7), retinoic acid (35), and estrogen (36) can drives CD4^+^CD25^−^ naïve T cells developing to CD4^+^CD25^+^ iTreg cells. Naringenin has been shown to induce iTreg cells from CD4^+^ T cells independent of TGF-β (37). In accordance with this, current study demonstrated that naringenin dose-dependently induced iTreg cells from anti-CD3/CD28 activated T cells (Supplemental Figure 1). In the presence of TGF-β, naringenin could further potentiate naïve CD4^+^ T cell conversion into iTreg cells. The mechanism of TGF-β-induced generation of Foxp3 is partly due to Smad proteins, such as Smad2 and Smad3 phosphorylation, activation, nuclear translocation, and finally, binding to the Foxp3 locus and causing Treg polarization (38, 39). Naringenin has been regarded as the Smad3 specific inhibitor via suppressing TGF-β ligand-receptor interaction (40, 41). Indeed, naringenin *in vitro* slightly inhibited Smad2 and Smad3 phosphorylation which results in decreased generation of Foxp3. However, naringenin promotes, rather than inhibits, iTreg cell differentiation. These contradictory observations remain to be further elucidated.

Notably, TGF-β enables naïve CD4^+^ T cells to become Th17 cells when co-cultured with pro-inflammatory cytokines, such as IL-6 (3). Increased IL-6 could redirect TGF-β-induced Treg differentiation toward Th17 cells and as such, tilts the Th17 and Treg balance. Since we found that naringenin inhibited Th17 differentiation and also diminished IL-6-induced suppression in iTreg development, we addressed whether naringenin exerted these effects by affecting IL-6 signaling. IL-6 signaling is mediated via binding to its two receptors: mIL-6R and sIL-6, which elicit classical IL-6R signaling and IL-6 *trans*-signaling, respectively. Naïve T cells have high mIL-6R expression that will be lost during inflammation (19). Of note, naringenin partly prevented the reduction of mIL-6R in activated T cells, followed a decrease in sIL-6R levels in cultured mediums, which could be generated by activated T cells through shedding of mIL-6R (20). These studies justifies our results given that, after naïve T cells were polarized under Th17 differentiated condition, naringenin prevented a decrease in mIL-6R, while decreased sIL-6R in cultured supernatants. Since we did not observe any difference in mgp130 between naringenin and control, it may be suggested that naringenin might inhibit IL-6 *trans*-signaling. This inhibited IL-6 signaling by naringenin was further verified to be functionally relevant as we showed that naringenin suppressed IL-6-induced STAT3 phosphorylation. In addition to phosphorylation, STAT3 activation can be regulated by acetylation on lysine 685, which promotes Th17 development (42). Our observation that STAT3 acetylation was inhibited by naringenin further support involvement of altered STAT activation in naringenin's effect.

Elevated sIL-6R by auto-reactive CD4^+^ T cells contributes to autoimmune disease development via conferring IL-6 responsiveness (20) as well as blocking Treg development (43). Combination with the observed impact of naringenin on IL-6 signaling in CD4^+^ T cell differentiation, we speculated that naringenin's benefits on EAE might be partly due to naringenin's effect on IL-6 signaling. To confirm this, we conducted relevant analysis using the samples from our *in vivo* studies. Consistent with our previous study, EAE mice had two-fold higher plasma sIL-6R levels compared to the healthy control mice and this increase in plasma sIL-6R was prevented by dietary naringenin, which is in agreement with the findings in the current *in vitro* study. Together with the observation in that *in vivo* study that naringenin reduced plasma IL-6 levels in EAE mice, these results suggest that the results from the current *in vitro* study are relevant to the *in vivo* situation and that naringenin may block IL-6 *trans*-signaling, at least in part by reducing IL-6 and sIL-6R levels. Next we further analyzed the plasma sgp130, a natural inhibitor of IL-6 *trans*-signaling, from naringenin-treated EAE mice. Naringenin prevented the decrease in plasma sgp130 in EAE mice. This is in agreement with the decreased plasma IL-6 and sIL-6R levels in naringenin-treated EAE mice because reduced IL-6/sIL-6R complex formation in the trans-signaling would be assumed to spare some sgp130.

In addition, IL-2, a T cell growth factor, has been demonstrated to inhibit Th17 development while promote Treg development (44, 45). Our previous study has shown that naringenin inhibited IL-2/IL-2R signaling pathway (10), which indicates that naringenin might promote Th17 generation and inhibit Treg development via modulating IL-2/IL-2R signaling in differentiating CD4^+^ T cells. However, naringenin actually inhibited Th17 generation while promoted iTreg development in the current study. Furthermore, Blimp-1, a key regulator of terminal differentiation in B cells and T cell linage, can repress IL-2, IFN-γ, and IL-17 and maintain Treg cell function (46–48). The underlying mechanisms are mediated by binding to their regulatory factors such as ifnγ, tbx21, bcl6, stat3, stat5, il17. However, whether these genes are involved in the effects of naringenin on CD4^+^ T cell differentiation is still unclear. Thus, we will plan a specific in depth study in the soon future to determine the role of IL-2/IL-2R signaling and these regulatory genes in naringenin's effect on CD4^+^ T cell differentiation involving altered Treg/Th17 balance and Th1 differentiation.

In summary, this study demonstrated that naringenin inhibited Th1 and Th17 development; while naringenin did not affect Th2 cells in IL-4 production, it decreased IL-10 and IL-13 production. In addition, naringenin promoted iTreg development and prevented IL-6-induced suppression on iTreg development, which may be associated inhibition of Th17 differentiation. To our knowledge, this is the first comprehensive study reporting that naringenin modulates functions of effector CD4^+^ T cell subsets via targeting their respectively transcription and/or transducer factors. Especially, inflammatory cytokine IL-6 signaling appears to be a key factor through which naringenin favorably influences the balance between Th17 and Treg cells, leading to an alleviated autoimmunity. These novel observations allow us to gain a better understanding for the mechanisms underlying the naringenin's beneficial effect in attenuating T-cell mediated autoimmune disorders. We propose that these effects of naringenin may have translational value in potential clinical application to prevent/mitigate T cell-mediated autoimmune diseases.

## Author contributions

JW and DW designed the research. XN, CW, and JW conducted research and analyzed data and interpreted the data. JW and DW wrote the paper. All authors reviewed the manuscript.

### Conflict of interest statement

The authors declare that the research was conducted in the absence of any commercial or financial relationships that could be construed as a potential conflict of interest.
