# Supplementary material for: Naringenin Modifies the Development of Lineage-Specific Effector CD4+ T Cells
Source: Front Immunol. 2018 Oct 1;9:2267. doi: 10.3389/fimmu.2018.02267 (PMC6174281; doi:10.3389/fimmu.2018.02267)
Supplement: Supplementary file 1 [file Data_Sheet_1.doc]

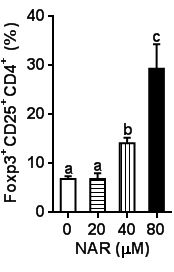


**Supplemental Fig. 1**. Effect of Naringenin on Treg cells from anti-CD3/CD28 activation. LN cells from naïve mice were stimulated with anti-CD3/CD28 in the presence of naringenin at the indicted concentrations for 72h, and CD4^+^CD25^+^Foxp3^+^ Treg cells were measured by flow cytometry after gating CD3^+^CD4^+^CD25^+^ T cells. Bar figures are mean ± SD of three independent experiments. Means without a common letter significantly differ at least at *P* < 0.05. NAR, naringenin.
